# Does fluctuating asymmetry of wing traits capture relative environmental stress in a lepidopteran?

**DOI:** 10.1002/ece3.7097

**Published:** 2021-01-11

**Authors:** Cole Symanski, Richard A. Redak

**Affiliations:** ^1^ Department of Entomology University of California at Riverside 900 University Ave Riverside CA 92521 USA; ^2^ Present address: Irvine CA USA

**Keywords:** developmental stress, fluctuating asymmetry, *Vanessa cardui*, viability, wing traits

## Abstract

1. Fluctuating asymmetry (FA) is hypothesized to be a useful predictor of population canalization, especially for organisms at risk from environmental change.
2. Identification of traits that meet statistical criteria as FA measures remains a challenge.
3. Here, a laboratory experiment subjected immature butterflies (*Vanessa cardui*) to diet and temperature conditions of varying stress levels. Variation in dietary macronutrient ratio (protein: carbohydrate) and rearing temperature (optimal: 25°C; elevated: 32°C) was introduced as stressors. Temperature and nutrition are key variables influencing ectotherm growth and fitness and so are likely to be important stressors that influence FA.
4. Individuals subjected to stressful conditions were predicted to show elevated FA of three wing size traits, as well as increased mortality and decreased adult body size.
5. Trait FA did not vary across treatments. Instead, treatment levels impacted viability: The combined incidence of pupal death and expression of significant wing malformations increased in treatment levels designated as stressful. Variation in adult dry mass also reflected predicted stress levels. Results suggest that individuals predicted to display increased FA either died or displayed gross developmental aberrations.
6. This experiment illustrates important constraints on the investigation of FA, including selection of appropriate traits and identification of appropriate levels of stressors to avoid elevated mortality. The latter concern brings into question the utility of FA as an indicator of stress in vulnerable, natural populations, where stress levels cannot be controlled, and mortality and fitness effects are often not quantifiable.

## Graphical abstract

While the topic of fluctuating asymmetry (FA) has received wide attention for its potential applicability to understanding stressors in populations, very few studies have examined this possibility rigorously both from an experimental perspective and an analytical one. This manuscript reports on a study that does both. In this study, FA was measured in the wings of a model butterfly.

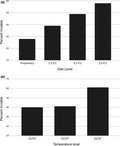

## 1 INTRODUCTION

In bilaterally symmetrical organisms, the clade that includes about 99% of all extant metazoan species (Freeman et al., 2014), fluctuating asymmetry (FA) of a trait is defined as deviation from perfect symmetry that is random in its direction (left‐ or right‐side biased) and normally distributed around a mean of zero (Palmer & Strobeck, 2003). Under favorable conditions, homeostatic mechanisms operating during development buffer effects of perturbations, a phenomenon often termed “developmental stability” (Gibbs & Breuker, 2006; Habel et al., 2012; Ludoski et al., 2014; Takahashi, 2018); effective buffering mechanisms result in low FA (Graham et al., 1998; Møller, 2006; Van Valen, 1962). Homeostatic mechanisms can be overwhelmed by a number of challenging environmental conditions, including poor diet quality (Windig & Nylin, 2002), food shortages (Stoks, 2000), high parasite density (Møller, 2006), and interference competition (Clark & McKenzie, 1992), as well as abiotic stresses including temperature (Gerard et al., 2018; Parsons, 1992; Sisodia & Singh, 2009). Biotic and abiotic “stressors” (Odum, 1985) lessen an organism's ability to allocate resources to development and reproduction (Beasley et al., 2013; Bilsma & Loeschcke, 2005). When stressors cause developing individuals to shift allocation away from maintaining developmental homeostasis to more pressing needs, such as ensuring survival (Møller, 2006), FA is expected to increase.

Fluctuating asymmetry has been a topic of interest to evolutionary and developmental biologists for many years (Ludwig, 1932; Van Valen, 1962; Waddington, 1942). More recently, ecologists and conservation biologists have advanced FA as a potentially powerful tool for measuring effects of environmental stressors such as increasing temperatures that result from anthropogenically induced climate change (Beasley et al., 2013; Lens & Eggermont, 2008), as well as a potential tool to identify populations at risk of extinction due to increased environmental stress (Leary & Allendorf, 1989). FA may be a useful surrogate for traditional methods of measuring population performance, which rely on labor‐intensive methods or destructive sampling (Clarke, 1995; Lens & Eggermont, 2008; Schmeller et al., 2011). Additional advantages include that FA measurements can be compared among individuals that are spatially and/or temporally isolated (Hogg et al., 2001), including comparisons of long‐dead museum specimens with living individuals (Schmeller et al., 2011). Several studies on mammals and birds have correlated the amount of FA with ecological indicators of stress, including degree of habitat fragmentation (Anciaes & Marini, 2001) and proximity to range limits (Auffray et al., 1999; Møller, 1997). However, studies on lepidopterans have reported mixed results in this regard (Kark et al., 2004; Windig et al., 2000; but see Benítez et al., 2015; Betzholtz, 2000).

Several criticisms have been leveled against studies of FA, notably concerning statistical design questions. Measurement error is an unavoidable concern for FA studies (Leung & Forbes, 1996; Lens et al., 2002; Palmer & Strobeck, 2003) because the effect size of FA is small relative to trait size and can be similar in magnitude to measurement error, especially in small organisms (Lens et al., 2002). Confirmation bias by scientists measuring FA who are not blind to sample population status may also contribute significantly to measurement error (Kozlov & Zvereva, 2015). Since an organism's traits vary in degree of FA in response to a given stressor (Polak et al., 2004), multiple traits should be included to estimate the overall stressor's impact, yet several studies have included only a single trait (Lens & Eggermont, 2008). Fortunately, Palmer and Strobeck (2003) developed a detailed set of guidelines to avoid these and related problems, and their guidelines have been closely adhered to in this study.

The purpose of this experiment was to investigate whether environmental stressors influence FA of the painted lady butterfly (*V cardui* L. [Lepidoptera: Nymphalidae]) under controlled laboratory conditions in which determination of fates of all subjects was possible. Temperature and diet manipulations were selected as stressors, as it is widely agreed that these are very influential determinants of individual survival and reproduction as well as distribution of many heterotrophic species (Boersma et al., 2016; Clissold & Simpson, 2015; Cross et al., 2015; Kim et al., 2020; Sørensen et al., 2016). A high temperature stressor was selected because of its relevance to global climate change, to which ectotherms may be particularly vulnerable (Burraco *et al*., 2020; Deutsch et al., 2008; Huey & Berrigan, 2001; Huey & Kingsolver, 1989). Evidence suggests that ectotherms tend to lack capacity to physiologically adjust maximum temperature tolerance (Gunderson & Stillman, 2015), and thermal performance curves of insects and other ectotherms decelerate rapidly above an optimal plateau (Clissold & Simpson, 2015; Deutsch et al., 2008; Sutton et al., 2018). These lines of evidence suggest that FA induced by temperature stress may be detectable at temperatures only slightly elevated above an optimal range.

Diet was included as a potential stressor because larval availability of macronutrients, especially the balance between carbohydrate and protein, has been shown to have important performance effects in holometabolous insects, impacting growth and development, adult survival, and reproduction (Andersen et al., 2010; Clissold & Simpson, 2015; Joern & Behmer, 1997; Kim et al., 2019, 2020; Roeder & Behmer, 2014; Simpson & Raubenheimer, 2012). Moreover, the protein needs of larval butterflies have been demonstrated (Behmer, 2009; O’Brien et al., 2004). These two stressors (temperature and diet) are natural companions because studies have shown that they exert reciprocal impacts: Macronutrient composition can influence tolerance to thermal stress, for example (Andersen et al., 2010; Kutz et al., 2019), and increases in temperature associated with climate change may influence availability of nutrients (Bauerfeind & Fischer, 2013; Rosenblatt & Schmitz, 2016). Finally, diet and temperature may interact to influence performance measures such growth and development rate, with specific optima varying among species and performance measures (Clissold & Simpson, 2015; Lee & Roh, 2010; Parsons & Joern, 2014). In this study, we did not make explicit predictions about interaction effects between stressors (see below), but we anticipated that the simultaneous presence of multiple stressors might increase phenotypic effects displayed by organisms.

Here we used the painted lady (*Vanessa cardui*) as an experimental model to measure three potential outcomes of temperature and diet stress: increased mortality of pupae/emerging adults, increased FA of adult wing traits, and decreased adult body mass. While our primary aim was to investigate FA, increased mortality and decreased body mass are alternative possible outcomes of stressors that have been previously identified (e.g., Andersen et al., 2010; Clissold & Simpson, 2015; Kim et al., 2020; Rosa & Saastamoinen, 2017). Where stress causes high mortality and deformity (“inviability”), individuals most likely to display high FA are also those most likely to die before completing development (Polak et al., 2004). Thus, where inviability occurs, patterns of FA may not show predicted effects because FA can only be measured in intact adults. Our study hypotheses were that (H1) developing organisms exposed to diet/temperature conditions that deviate from those under which captive individuals typically thrive would display phenotypic consequences consistent with increased developmental stress; and that (H2) when developing organisms are exposed to stressors for longer intervals, increased stress results in greater phenotypic consequences for adults.

The painted lady is an appropriate laboratory model because a commercial stock, captive bred under known conditions (“lab‐adapted stock”), is readily available and is easy to culture. This sexually monomorphic species, with perhaps the broadest distribution of any butterfly (Opler & Krizek, 1984; Poston et al., 1977), is a generalist herbivore that feeds on 100 + species from at least 25 families (Janz, 2005), including numerous cultivated crops (Zhang, 1994); it is not known to utilize secondary plant compounds (VanOverbeke et al., 2017). The species can be reared on artificial diets derived for generalist lepidopterans (Ahmad et al., 1989; VanOverbeke et al., 2017), and research has shown that a lab‐adapted stock thrives on a diet developed by a biological supply house (Ellis & Bowers, 1998). Researchers have reported attempting to laboratory rear immatures at temperatures ranging from 12 to 33°C (Hammad et al., 1972; Kelly & Debinski, 1999; Kennelly et al., 2017; Poston et al., 1977), noting increased mortality above 30° (Kennelly et al., 2017; Poston et al., 1977; and see Section 2), and developmental failure at low temperatures (below 18°C; we have not found reports regarding the temperature range under which immatures occur in nature). The dietary breadth and temperature range identified for successful rearing (circa 21–28°C) contribute to this species’ ease of use in the laboratory and amenability to experimental manipulations.

Six sets of predictions were made at the outset of the study. Prediction set A is that individuals raised under temperature and diet conditions mirroring those typically experienced by lab‐adapted stock will display the least FA as adults. They will also display the largest body size and lower mortality than individuals reared on chemically defined diets (diets with known macronutrient constituents and no unknown additives). Prediction set B is that elevated temperature will result in increased FA of adult wing traits and/or decreased adult dry mass (dry mass is a proxy for body mass). Prediction set C is that elevated temperature during the larval stage only will result in less adult FA than does elevated temperature throughout development (larval + pupal stages). The rationale for this expectation is that longer intervals of stress should result in greater phenotypic deviations than shorter ones. Since pupae do not feed and much is unknown about the costs of pupal metamorphosis, no prediction was made about mass differences between individuals that experienced elevated temperature during the larval stage versus the entire immature period. Increased temperature was not predicted to impact mortality because we selected a temperature stressor that preliminary data suggested would not greatly affect morality in order to maximize opportunity to detect FA. Prediction set D is that individuals reared on (chemically defined) diets other than the commercial painted lady diet will have greater FA and/or mortality and/or lower dry mass than those reared on the commercial diet. This prediction is based on the assumption that the commercial diet was developed over time to improve butterfly performance and knowledge that the captive stock had had multiple generations to adapt to the diet. Also, the chemically defined diets selected as stressors are likely deficient in nutrients supplied by the commercial diet. Prediction set E is that the chemically defined diet that leads to the greatest FA/mortality and/or lowest adult mass is the 1:3 protein: carbohydrate diet. These predictions are based on previous research from this laboratory (VanOverbeke, 2011; VanOverbeke et al., 2017) and another (Ellis & Bowers, 1998) using chemically defined diets with this species, as well as literature on effects of high carbohydrate diets on larval butterflies (Behmer, 2009; O’Brien et al., 2004). Prediction F is that, within treatment groups, individuals that survive past eclosion will have higher dry mass than those that do not.

## 2 MATERIALS AND METHODS

### 2.1 Butterfly culture and experiment initiation

Butterflies were obtained as five separate shipments (100 eggs each) from a commercial supplier (Carolina Biological Supply: Burlington, NC, USA) that also supplied a proprietary rearing medium (Carolina Biological Painted lady culture medium, or “Carolina diet”). The commercial stock had been maintained on this diet under standard conditions for numerous generations, with periodic additions of wild‐caught butterflies. Purchased eggs were reared to adulthood on Carolina diet, and two generations were reared before the experiment was initiated.

The rearing protocol involved several steps. Flight cages (cylinders 0.91 m tall with a diameter of 0.61 m) that held 30 adults were supplied with food (sugar–honey water) and oviposition sites (*Achillea millefolium* L. [Asteraceae] flowers, refreshed every other day). Eggs were collected every 2 days, sterilized in a 5% bleach solution for 2 min, and placed in group‐rearing chambers on the Carolina diet. Chambers were inspected daily for pupae. Pupae were transferred to an eclosion box within 2 days of pupation. Within 24 hr of eclosion, butterflies were transferred to flight cages. Between generations, butterflies were randomly shuffled among flight cages to ensure outcrossing.

The F2 generation of eggs was produced in December 2015, at which time eggs were collected over a 48‐hr period from 10 flight cages containing 30 butterflies each. Upon collection, eggs were sterilized and transferred to rearing chambers as above, where they developed on Carolina diet for the first two instars. When 30 or more larvae simultaneously reached third instar, individuals were randomly assigned, one at a time, to one of 10 experimental treatments described below. A total of 600 s‐generation larvae were individually transferred with a paint brush into 1oz (Solo^®^) soufflé cups. These cups were then placed into their respective growth chambers, using a randomization design to assign individual larvae to shelves. Experimental conditions commenced immediately thereafter.

The experiment was designed to be replicated through the use of two sets of two growth chambers, the maximum number of chambers available. A fully factorial design could not be implemented because of space limitations in the available growth chambers, a constraint that was exacerbated by preliminary results indicating that a substantial sample size (30) was required for each treatment group per replication, due to mortality. A baseline treatment (F1: butterflies reared on a diet and temperature to which they were accustomed) was established as a comparison for results of experimental stressors (Table 2).

Unfortunately, one of the growth chambers malfunctioned, resulting in very high mortality. Accordingly, here we report results only for the first replicate experiment (600 larvae randomly assigned to treatment).

### 2.2 Stressors

Larvae were reared in growth chambers (Percival incubators—model 136LL) maintained on a L16: D8 photoperiod maintained at 25 ± 1°C, the other at 32 ± 1°C. Each chamber contained two shelves. Temperature levels were selected following a pilot investigation in which nearly complete larval mortality was observed at a constant rearing temperature of 35°C; by contrast, mortality at 32°C was only moderately elevated (10%) above that observed at 25°C (the temperature recommended by our commercial supplier and included within the optimal range by Poston et al. (1977)). Accordingly, 25°C was chosen as the “optimal” developmental temperature and 32°C was chosen as the “stressor” temperature. To investigate the prediction that individuals subjected to stressful temperatures throughout development would show higher FA than those stressed for only part of development, temperature treatment levels included elevated temperature (32°C) during both the larval and pupal stages (temperature level 3) and elevated temperature during the larval stage only (temperature level 2; Table 2); pupae were transferred between growth chambers 1 day after pupation.

As described above, the stock of *V. cardui* used here had been maintained on Carolina medium for a number of generations prior to the experiment, presumably becoming adapted to it. Therefore, Carolina diet was used here as the baseline (diet level 1). Three additional diet levels were employed to examine the effect of macronutrient ratio on induction of FA. These chemically defined diets were modified from previous recipes devised for *Vanessa* (VanOverbeke, 2011; VanOverbeke et al., 2017; see also Ahmad et al., 1989 for *Manduca sexta* L. [Lepidoptera: Sphingidae]) because preliminary results showed that many pupae failed to initiate or complete eclosion. Accordingly, linseed oil, a source of fatty acids (linoleic and linolenic acids) necessary for ecdysis in some insects (Nation, 2015), was added to diet recipes (Table 1). Diet treatments included equal amounts of protein and carbohydrate (1:1; diet level 2), as well as high‐protein (3:1; diet level 3) and low‐protein (1:3; diet level 4) ratios (Table 2). In sum, the experiment included a total of 10 treatments, generated from two factors: diet and temperature. There were three temperature levels and four diet levels (Table 2).

**Table 1:** Laboratory diet recipes used to feed 3rd through 5th instar larvae in this experiment^a^

Ingredients	Diet factor levels (FL)		
	1:1 P:C (FL2)	3:1 P:C (FL3)	1:3 P:C (FL4)
Water (ml)	850	850	850
Casein (g)	60	90	30
Sucrose (g)	60	30	90
Agar (g)	20	20	20
Wesson's salt (g)	14	14	14
Vitamin mixture (g)	10	10	10
Ascorbic acid (g)	5	5	5
Antibiotic (g)	4	4	4
Linseed oil (ml)	4	4	4
Cholesterol (g)	4	4	4
Kanamycin sulfate (g)	3	3	3
Methylparabenzoate (g)	2	2	2
Sorbic acid (g)	2	2	2
Choline chloride (g)	1	1	1

^a^ Differs from painted lady chemically defined laboratory diet used by Vanoverbeke (2011) in that no formaldehyde was included here, and linseed oil and kanamycin sulfate were not components of Vanoverbeke's diet. Linseed oil contains steroids important for eclosion.

**Table 2:** Description of treatments and diet and temperature levels

Treatment	Diet		Temperature	
	Level	Description	Level	Description
F1	1	Carolina painted lady diet	1	25°C from 3rd instar larva eclosion
F2	2	laboratory diet—1:1 protein: carbohydrate	1	25°C from 3rd instar larva eclosion
F3	3	laboratory diet—3:1 protein: carbohydrate	1	25°C from 3rd instar larva eclosion
F4	4	laboratory diet—1:3 protein: carbohydrate	1	25°C from 3rd instar larva eclosion
F5^a^	2	laboratory diet—1:1 protein: carbohydrate	2	25°C from 3rd instar larva pupation; thereafter, 32°C eclosion
F6	2	laboratory diet—1:1 protein: carbohydrate	3	32°C from 3rd instar larva eclosion
F7^a^	3	laboratory diet—3:1 protein: carbohydrate	2	25°C from 3rd instar larva pupation; thereafter, 32°C eclosion
F8	3	laboratory diet—3:1 protein: carbohydrate	3	32°C from 3rd instar larva eclosion
F9^a^	4	laboratory diet—1:3 protein: carbohydrate	2	25°C from 3rd instar larva pupation; thereafter, 32°C eclosion
F10	4	laboratory diet—1:3 protein: carbohydrate	3	32°C from 3rd instar larva eclosion

^a^ Treatments in bold face were moved from chamber 2 to chamber 1 one day after pupation in order to accommodate the change in environmental temperature.

### 2.3 Eclosion and processing of specimens

Starting 3 days after the first pupae developed, chambers were monitored every 12 hr for signs of imminent eclosion. Soufflé cups housing pupae were cleaned out (food and frass removed) or replaced to allow enclosing butterflies sufficient room to dry wings. Individuals were allowed to dry wings fully in the cups and were thereafter promptly moved to a cooler (18°C) growth chamber, where they were held for 24 hr; this procedure allowed butterflies to evacuate meconium while remaining sedentary and thus avoided wing damage. Butterflies were subsequently placed individually into glassine envelopes and euthanized in a freezer.

Frozen specimens were sorted into two categories: (a) those with obvious wing deformities and those that failed to fully escape their puparia versus (b) those that had fully developed wings. Butterflies in the first category were classified as “inviable,” as they would have been unlikely to survive and reproduce; their nondirectional wing asymmetries could not be measured. Butterflies in the second category were deemed “viable” and were included as subjects in the investigation of FA (Table 3).

**Table 3:** Summary of fates of experimental subjects and final sample sizes for analyses

Treatment	Diet level	Temp level	*N* assigned	*N* died	*N* damaged wings^a^	*N* inviable^b^	*N* viable/ measured	*N* Excluded^c^	*N* Included^d^
1	1	1	60	7	10	17	43	3	40
2	2	1	60	11	8	19	41	0	41
3	3	1	60	19	12	31	29	0	29
4	4	1	60	21	8	29	31	1	30
5	2	2	60	11	10	21	39	0	39
6	2	3	60	17	13	30	30	1	29
7	3	2	60	9	14	23	37	3	34
8	3	3	60	18	16	34	26	1	25
9	4	2	60	22	8	30	30	1	29
10	4	3	60	28	17	35	15	1	14
Total subjects			600	163	116	279	321	11	310

^a^ Damaged individuals had wings that failed to dry properly (both forewings and hindwings damaged).

^b^ Sum of *N*_died_ + *N*_damaged wings_

^c^ Individuals excluded from FA results because two or more traits met statistical criteria for exclusion and/or had handling or other damage that precluded measurement of traits.

^d^ Final sample size: number of individuals included in FA results (had at least two traits that were not excluded).

Specimens were subsequently rehydrated to make wings pliant for removal from the abdomen. Wings were cut at the abdomen base in the same order across all specimens (right forewing/left forewing/left hindwing/right hindwing). Wings of viable specimens were then sealed in left–right pairs in clear packaging tape for imaging. Scans of wings were taken on an HP printer (LaserJet M1522n) at 1,080 dp.

After dissection, the head, thorax, and abdomen of each specimen—both viable and inviable—were placed together in a glassine envelope and allowed to desiccate on the laboratory bench for 2 weeks. Total dry mass of these body parts was obtained using a NewClassic MF balance (Mettler Toledo model MS205DU). Abdomens were scored for the presence/absence of residual meconium.

### 2.4 Trait selection and measurement procedures

The experiment incorporated several criteria identified by Palmer and Strobeck (2003) for selection of traits in investigations of FA: (a) Multiple traits should be included and should display low phenotypic intercorrelation in the direction and size of their left–right side differences. (b) Selected traits must display left–right side variances significantly greater than that which is caused by measurement error and should display similar magnitudes of measurement error. (c) Candidate traits must be screened for the presence of other types of asymmetry; those that exhibit antisymmetry (bimodality) are not suitable. Below, we discuss initial trait selection, and in remaining Methods sections, we explain how the additional criteria were addressed, as well as the framework for statistical evaluation of FA that we used.

Preliminary investigations focused on seven candidate wing traits, including five area traits and two vector traits. Three of the area traits were wing spots, which are characteristic of many lepidopterans (*Pararge aegeria* L. [Nymphalidae]; *Parnassius apollo* L. [Papilionidae] & *Bicyclus anynana* Butler [Nymphalidae]) for which FA has been investigated (Gibbs & Breuker, 2006; Habel et al., 2012; Talloen et al., 2004). The two remaining area traits were forewing and hindwing areas; finally, the lengths of one forewing and one hindwing vein were included. These traits were measured (and later remeasured) in 50 randomly selected individuals that were used for measurement practice (i.e., they were not part of the experiment). The measurements made from the seven traits were analyzed for relative measurement error and for correlations among the left–right side differences (i.e., trait asymmetry). For four traits, differences in signed asymmetry were strongly intercorrelated, implying developmental interdependence (Palmer & Strobeck, 2003); these traits were dropped from further consideration. Three remaining traits—forewing area, hindwing spot 1 area (spot at the anal angle of the wing), and hindwing vein length (second branch of cubital vein)—were not strongly intercorrelated and did not differ in measurement error: These traits were used to investigate effects of stressors on wing trait FA.

**Figure 1:**
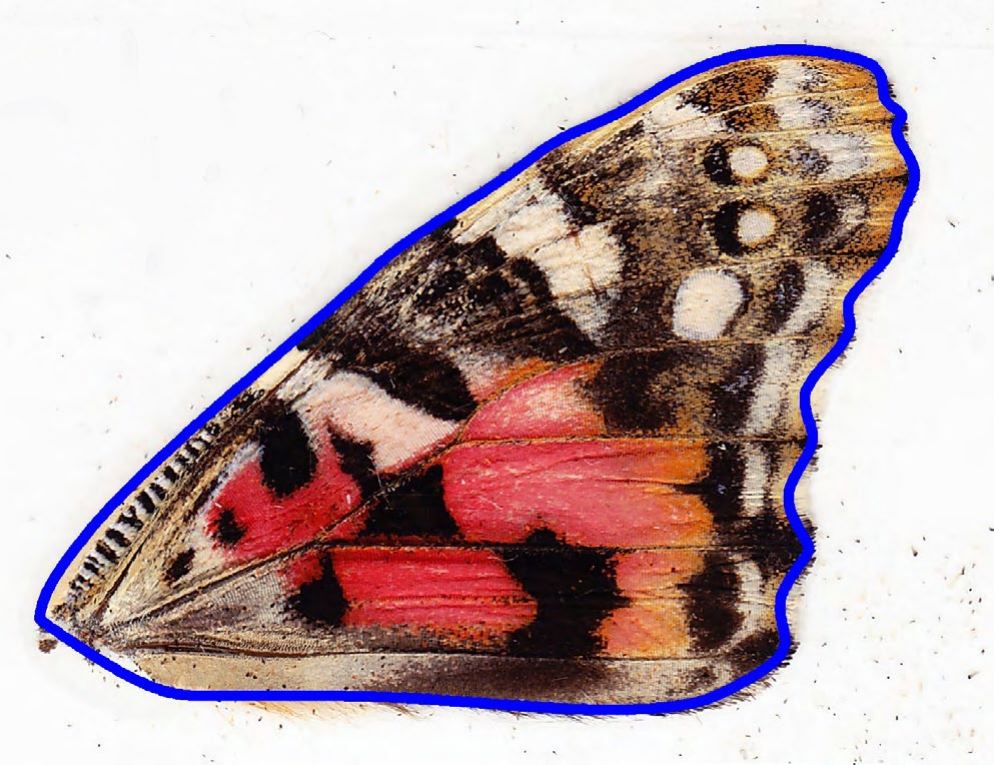
Illustration of trait forewing area. Ventral view of a left forewing. Area under the blue outline is an example of the variable forewing area. Note that the flange surrounding the perimeter of the wing was excluded from measurement. There is also a small negative space inside the polygon that was created erroneously when cutting the wing

**Figure 2:**
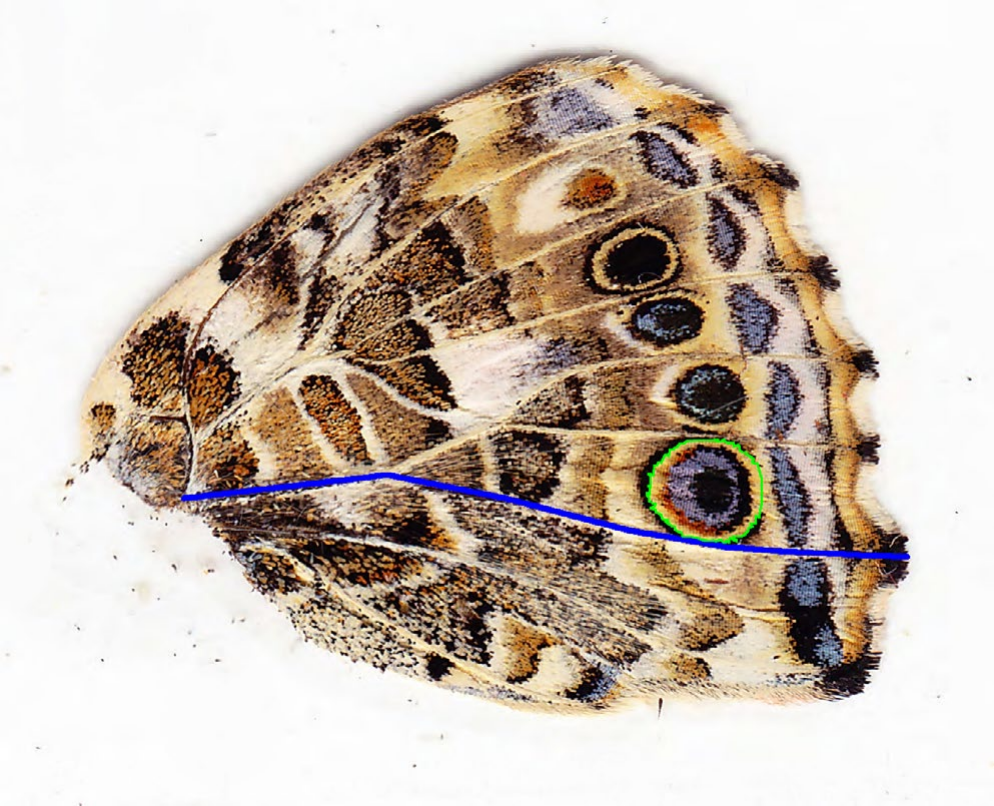
Illustration of traits hindwing vein length and hindwing spot 1 area. Ventral view of a left hindwing. The blue line is an example of the variable hindwing vein length. The green polygon is an example of the variable hindwing spot 1 area

Measurement of forewing area (100% image magnification) and hindwing spot 1 area (400% image magnification) was accomplished using the “polygon” tool in ImageJ (Schneider et al., 2012) to manually outline the border of these traits (see Figures 1 and 2). Hindwing vein length (100% image magnification) was measured, using the “segmented line” tool, as the shortest length from a starting point near the abdomen to the border of the flange at the outer edge of the wing (Figure 2).

About one‐third of the subjects were measured twice in order to quantify measurement error; the remaining individuals were measured one time. Individuals that were measured twice were selected using a stratified random design to ensure inclusion of approximately 10 individuals from each treatment. These individuals were first measured shortly after practice measurements were completed and remeasured after about two‐thirds of all subjects had been measured once.

### 2.5 Indices of FA

FA of individual traits was evaluated using the mean of the absolute value of the left side minus the right‐side (mean |L‐R|) trait measurement for twice‐measured individuals and |L‐R| size for individuals measured once, after establishing that this value was greater than measurement error and met various statistical criteria (named “FA1” in Palmer, 1994; Palmer & Strobeck, 2003).

An individual butterfly's composite FA (“FA11” in Palmer, 1994) was calculated by summing observed FA1 scores for individual traits. Only traits that had similar measurement errors in the full data set (forewing area [mm^2^] and hindwing vein length [mm]) were included in the composite index. Since traits differ in their absolute magnitude of FA, values were ln‐transformed prior to summing them so that traits contributed approximately equally to the composite FA index (Palmer & Strobeck, 2003).

### 2.6 Analyses

A total of 310 individuals were included in FA analyses; 101 of these were measured twice. Palmer and Strobeck’s (2003) protocol for analysis of trait FA was used in the analysis procedure described below. Data for individuals measured twice were analyzed first and were used to quantify measurement error. Under the assumption that measurement error was the same for individuals measured once, the remaining data analyses were conducted on the full data set.

#### 2.6.1 Screening for outliers

Two series of analyses screened the data set for problems that inflate the estimate of trait FA. The first set of analyses sought to identify cases of “bad” raw measurements in the replicated data set, where sample sizes ranged from 10 [diet level 1] to 46 [temperature level 1]. The category of “bad” raw measurements includes instances in which larger than usual error was made in measuring a specimen, as well as errors unrelated to measurement precision per se (such as data entry errors). The second set of analyses was performed to identify possibly “aberrant” individuals, that is, those that are outliers for asymmetry of one or more traits; these analyses were conducted both on the replicated data set and the full data set. Analyses were initially performed for each diet and temperature level (seven separate analyses for each of three traits). The rationale for performing analysis on each factor level is that if outliers are disproportionately represented in one or two levels, they would be difficult to detect in the aggregate data set, yet could potentially bias outcome of FA analyses of the full data set (Palmer & Strobeck, 2003). Analyses involved construction of scatterplots of the data for visual identification of outliers, followed by application of either Dixon's test (when *N* ≤ 25) or Grubb's test (when *N* > 25) to ascertain their statistical significance. Because data inspection involved multiple groupings of the data (four diet levels; three temperature levels), sequential Bonferroni corrections were made to the *p*‐values obtained from these analyses (Palmer & Strobeck, 2003).

When a data point was found to be statistically significant as a “bad measurement,” two new measurements were taken on separate days, and these replaced the original measurements. When a specimen was deemed to be significantly “aberrant” for a single trait, its values for that trait were dropped from the data set. Such outliers are likely caused by aberrant developmental programming and are not representations of FA (Palmer, 1994). When more than one trait of a given individual was found to be an outlier for asymmetry, all measurements for that individual were dropped from the data set. This procedure was implemented after discovery that about half the individuals found to be outliers for one trait were also outliers for at least one other trait.

After completion of data inspection for each of the seven levels, the data set (replicated or full) was aggregated and the steps outlined above repeated (Palmer & Strobeck, 2003).

During the screening process for the replicated data set, three individuals were found to be statistically significant outliers for [L‐R] differences in two or more traits and were dropped from the study. For three additional individuals, a single trait showed anomalous asymmetry; in these cases, values for the outlier trait were dropped, but the individuals remained in the data set. In addition, human measurement error was detected and corrected, in three measurements. In the full data set, five additional specimens were found to be significant outliers for two or more traits and four additional specimens were outliers for a single trait.

#### 2.6.2 Departures from “ideal” FA

Following elimination of outliers, a series of two‐way, mixed‐model ANOVAs was performed on the replicated data set to test whether small nondirectional asymmetries (asymmetries random in their direction, but centered around zero) were greater than measurement error for each trait. These tests also examined whether traits showed directional asymmetry (one side consistently larger than the other). When directional asymmetry is present, estimates of FA are artificially inflated. Therefore, such asymmetry was corrected by adding half the value of the mean difference between sides to the smaller side and subtracting the same amount from the larger side (Palmer, 1994). In these ANOVAs, the dependent variable was trait size (e.g., forewing area), and the independent variables were body “side” (L or R; a fixed factor) and “individual” (a random factor). These analyses were initially performed on each diet and temperature level (seven separate analyses on sample sizes that varied between nine and 44) and later repeated on the aggregate data set. Analysis was performed on each treatment level as a precaution against the possibility that relative measurement error varied among treatments due to treatment variation in occurrence of subtle deformities (Palmer & Strobeck, 2003).

The two‐way ANOVAs to evaluate relative magnitude of asymmetries of each trait performed for each diet, and temperature level of the replicated data set showed that nondirectional asymmetry was significantly greater than measurement error in all cases. Parallel tests for directional asymmetry were significant for five of seven comparisons involving hindwing vein length, but not significant in analyses of forewing area and hindwing spot area. After correction for the average [L‐R] hindwing vein length difference (0.1017 mm), the ANOVA procedure was repeated on the pooled data set. At this point, all three traits exhibited highly significant nondirectional asymmetry, and directional asymmetry was no longer significant for any trait. In the full data set, the average [L‐R] hindwing vein length difference was 0.06 mm; this number differed significantly from zero (*t* = −4.00, *N* = 308, *p* = .001), so a correction for directional asymmetry was applied.

#### 2.6.3 Trait differences in measurement error

To determine whether the three traits differed in measurement error in the replicated data set, correction for differences in trait size was carried out by ln transformation of raw measurements. Then, Levene's test of homogeneity of variances (Brown & Forsythe, 1974) was performed on the aggregate replicated data set (*N* = 101) to compare the absolute values of the second versus first set of ln‐transformed measurements for each trait; these tests were performed using the STATA^®^ “robvar” command. Similarity of measurement error is necessary in order to combine indices of FA of individual traits into a composite index.

Measurement error significantly differed among traits: Specifically, the average absolute difference between the second set of measurements versus the first was about twice as great for hindspot area (mean ± *SE*: 0.064 ± 0.0049 mm^2^) than either forewing area (0.0032 ± 0.0024 mm^2^) or hindwing vein length (0.0034 ± 0.0027 mm; Levene's test, *W* = 12.468, *df* = 2, 299, *p* < .0001). No differences in measurement error were found as a function of diet level (*W* = 0.183, *df* = 3,298, *p* = .91) or temperature level (*W* = 0.619, *df* = 2, 299, *p* = .54).

Remaining analyses were performed on the complete data set (*N* = 310).

#### 2.6.4 Relationship between magnitude of trait asymmetry and trait size

Spearman tests were used to assess whether the amount of asymmetry |L‐R| of a given trait was correlated with trait size. This step is important because the existence of a positive correlation is problematic for interpretation of significance of asymmetry (Palmer, 1994). Neither forewing area (Spearman rho = 0.05, *N* = 303, *p* = .39) nor hindwing vein length (rho = −0.006, *N* = 308, *p* = .92) displayed a correlation between the absolute size of trait asymmetry and trait size. A weak positive correlation was found for hindwing spot 1 area (rho = 0.12, *N* = 295), but this was not significant (*p* = .12) after Bonferroni correction.

#### 2.6.5 Inspection of trait distributions

For traits to be used as estimates of FA, they should exhibit a normal distribution, with a mean centered near zero (Palmer & Strobeck, 2003). Tests for departures from these criteria included examination for antisymmetry (bimodality), skewness, and kurtosis. Skewness and kurtosis were statistically evaluated using the skewness–kurtosis test in STATA^®^. One‐sample *t* tests were used to ascertain departure from a mean asymmetry value of zero. Since each trait was subjected to three statistical tests for departure from normality, sequential Bonferroni tests were applied in the evaluation of whether trait asymmetry distributions deviated from normality (Palmer & Strobeck, 2003). Forewing area and hindwing vein length were normally distributed, while the distribution of hindwing spot 1 area showed significant kurtosis (*p* = .014 after correction for multiple comparisons). Accordingly, subsequent analyses and tests of predictions were performed only for forewing and hindwing vein length.

#### 2.6.6 Treatment effects on FA

Trait asymmetries that were found to be normally distributed and for which the magnitude of asymmetry was not correlated with trait size were deemed measures of FA. Linear mixed models were then applied to ask whether FA of single traits (“FA1”) or the trait composite (“FA11”) differed among treatments. In these tests, a measure of FA was the dependent variable, and diet and temperature were included as fixed factors, while rearing shelf (top or bottom) was a block. An equivalent linear mixed model was performed to assess influence of treatments on dry mass. Throughout, nonsignificant interaction terms were dropped. When parametric analysis generated significant residuals, the validity of the linear mixed model was called into question. Also, because the inclusion of subjects reared on the Carolina diet (diet level 1) at only one temperature resulted in an unbalanced design, the statistical routine could not produce an overall error term for the linear mixed models. For these reasons, nonparametric analyses of variance (Kruskal–Wallis tests) were also performed to provide comparison.

#### 2.6.7 Additional tests

For the 600 subjects that entered the experiment, Pearson's *C* ^2^ tests were used to determine whether diet or temperature levels had differential effects on viability (completion of eclosion with undamaged wings). Analyses determined whether the proportion of inviable specimens varied as a function of diet or treatment level.

All analyses were two‐tailed and were performed in STATA 14^®^ (StataCorp LP).

## 3 RESULTS

Overall, 53.5% of the experimental population survived eclosion with undamaged wings; these individuals were measured for trait asymmetry. A substantial fraction of the population failed to survive through eclosion (27.2%), and an additional fraction had wing deformities inconsistent with survival in nature (19.3%). (See Table 3 for sample sizes by treatment level.)

### 3.1 Treatment effects on FA

None of three linear mixed models to determine whether diet and temperature levels predicted FA of forewing area or hindwing vein length (FA1) or composite trait (forewing area + hindwing vein length) FA (FA11) was significant. No interaction between factors was significant, so the interaction term was removed before reporting the mode. Shelf effects were also nonsignificant (Table 4). Since these parametric models generated highly significant residuals, results were supplemented by nonparametric analyses. None of the six Kruskal–Wallis tests showed a significant influence of either diet or temperature levels on any FA measure (Table 5). Means (±*SE*) of FA for all levels of both factors are reported in Table 6.

**Table 4:** Influence of diet and temperature factors on FA1 and FA11 in the full data set (linear mixed models, after interaction term removed, ^a^with shelf as a random effect; no corrections for multiple comparisons)

Dependent variable	*N*	Wald *C* ^2^	Model *p*	Diet effect *p*	Temperature effect *p*
Forewing area FA1^b^	303	6.46	.26	.23	.72
Hindwing vein length FA1^b^	308	5.10	.40	.21	.63
Composite FA11^b^	301	4.29	.51	.25	.75

^a^ Variance contribution of shelf effect approached zero in all models.

^b^ Model residuals are highly significant.

**Table 5:** Nonparametric models for effects of diet and temperature on FA1 and FA11 (Kruskal–Wallis tests) for the full data set (no corrections for multiple comparisons)

Dependent variable	*N*	Diet effect			Temperature effect		
		*C* ^2^	*df*	*p*	*C* ^2^	*df*	*p*
Forewing area FA1	303	3.473	3	.32	1.829	2	.40
Hindwing vein length FA1	308	1.810	3	.61	1.966	2	.37
Composite FA11	301	3.557	3	.31	1.902	2	.39

**Table 6:** Descriptive statistics of FA scores by factor level for full data set (raw means)

Factor	Level	Variable	Mean ± *SE*	*N*
Diet	1	FW FA1	5.66 ± 0.72	40
	1	HV FA1	0.25 ± 0.04	39
	1	FA11	0.027 ± 0.002	39
	2	FW FA1	4.09 ± 0.31	106
	2	HV FA1	0.21 ± 0.01	108
	2	FA11	0.021 ± 0.001	105
	3	FW FA1	4.59 ± 0.44	86
	3	HV FA1	0.21 ± 0.02	88
	3	FA11	0.024 ± 0.002	86
	4	FW FA1	4.17 ± 0.40	71
	4	HV FA1	0.19 ± 0.02	73
	4	FA11	0.023 ± 0.001	71
Temperature	1	FW FA1	4.76 ± 0.33	138
	1	HV FA1	0.21 ± 0.01	138
	1	FA11	0.023 ± 0.001	136
	2	FW FA1	4.04 ± 0.34	100
	2	HV FA1	0.19±0.02	102
	2	FA11	0.023 ± 0.002	100
	3	FW FA1	4.45 ± 0.48	65
	3	HV FA1	0.022 ± 0.02	68
	3	FA11	0.024 ± 0.002	65

### 3.2 Additional tests

#### 3.2.1 Viability

**Figure 3:**
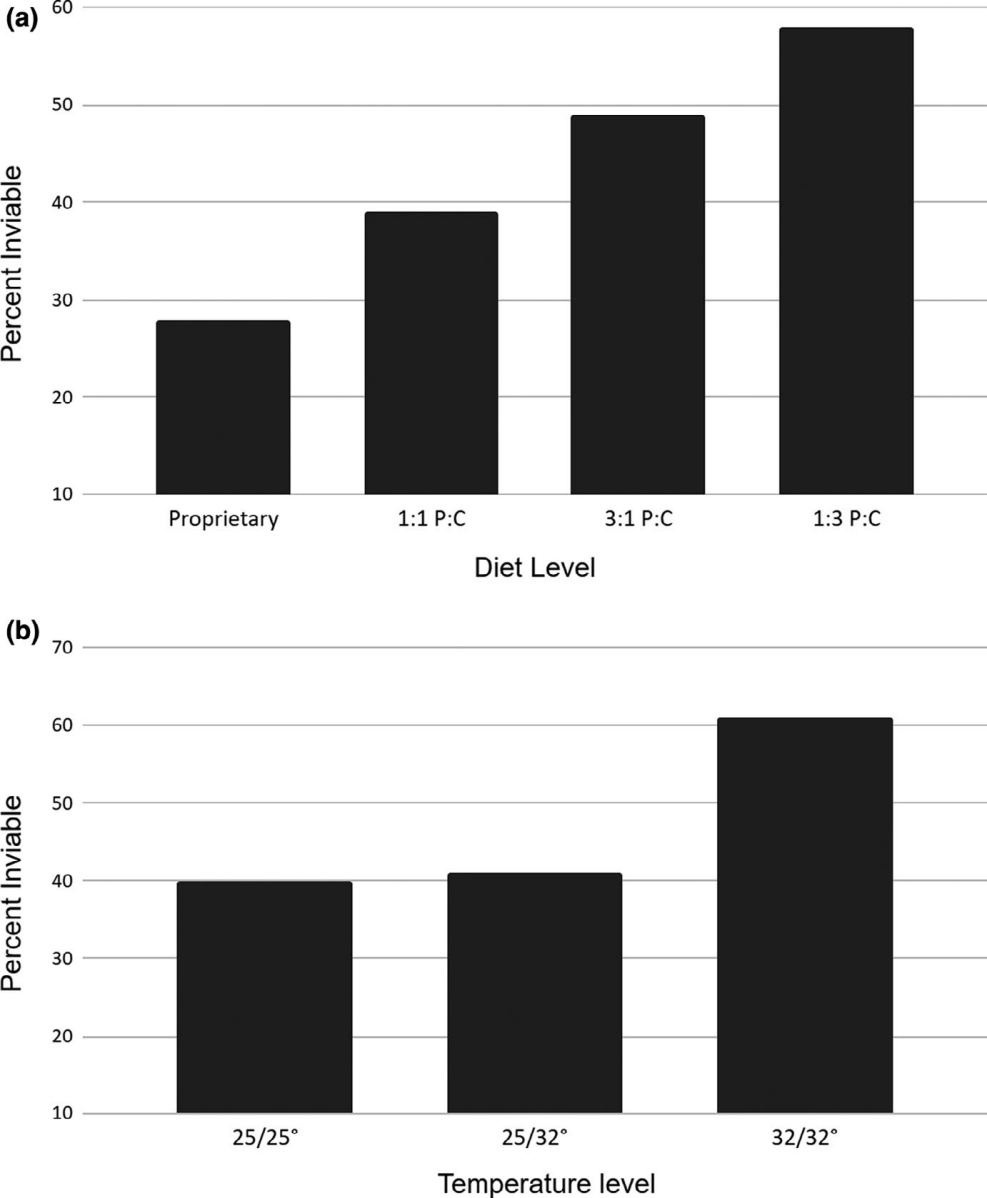
Inviability (mortality + significant damage) as a function of diet (a) and temperature (b)

Frequency of inviability (specimens that failed to survive eclosion and those with substantial wing defects: Table 3) differed on the basis of diet level (*C* ^2^ = 14.764, *df* = 3, *p* < .005) and temperature level (*C* ^2^ = 10.952, *df* = 2, *p* < .01 after correction for multiple comparisons). Post hoc analyses indicated that viability was highest on the Carolina diet (*p* < .025) and was lowest among specimens reared on a low‐protein diet (diet level 4: *p* < .025; Figure 3a). Viability was reduced in subjects that experienced elevated temperature throughout development (temperature level 3: *p* < .01, Figure 3b).

#### 3.2.2 Dry mass

**Figure 4:**
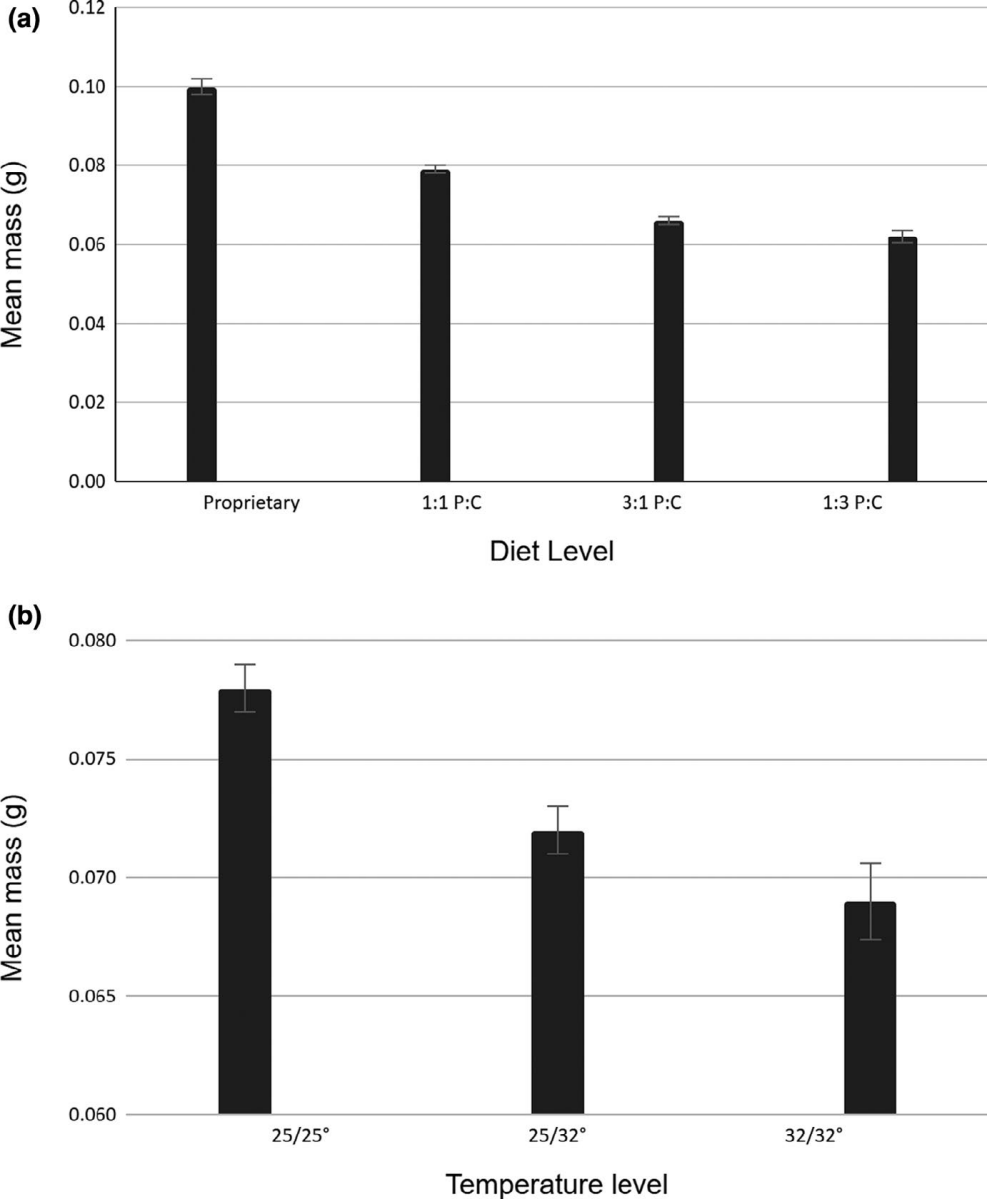
Dry mass (mean ± *SE*) as a function of diet (a) and temperature (b). (a) All a posteriori comparisons of the dry mass means of proprietary diet, the 1:1 protein: carbohydrate diet, and the 3:1 protein: carbohydrate diet were highly significant (*p*’s < .0001); the comparison between the 3:1 and 1:3 diet ratios was also significant (*z* = −2.32, *p* = .021). (b) Mean dry mass differed between butterflies reared at 25°C throughout development and other factor levels (*p*’s < .0001), but not between the higher temperature factor levels (*z* = −1.47, *p* = .14)

The linear mixed model predicting dry mass of viable specimens was highly significant (Wald *C* ^2^ = 355.51, 297 observations, 2 blocks, *p* < .0001). Both diet (*C* ^2^ = 238.56, *df* = 3, *p* < .0001) and temperature (*C* ^2^ = 24.70, *df* = 2, *p* < .0001) made significant contributions to the model. Dry mass differed significantly among all diet levels: Butterflies reared on the Carolina diet had the greatest dry mass, and those on the low‐protein diet had the lowest (Figure 4a). Dry mass of butterflies reared at 25°C throughout development (temperature level 1) was greater than that of butterflies exposed to higher temperatures as larvae. However, no mass difference was observed between butterflies maintained at 32°C throughout development (temperature level 3) and those that experienced a 25°C environment as pupae (temperature level 2; Figure 4b).

The linear mixed model exploring factor effects on dry mass of specimens classified as inviable showed the same pattern as found for viable specimens (Wald *C* ^2^ = 120.73, 83 observations, 2 blocks, *p* < .0001), with both significant diet (*C* ^2^ = 82.65, *df* = 3, *p* < .0001) and temperature (*C* ^2^ = 6.49, *df* = 2, *p* = .039) effects; specimens with residual meconium weighed on average 13.4% more (marginal mean ± *SE*: 0.0694 ± 0.002 g) than those that had expelled their meconium before death (marginal mean ± *SE*: 0.0612 ± 0.002 g; *C* ^2^ = 8.32, *df* = 1, *p* = .004).

Finally, a linear mixed model was performed to determine whether inviable individuals tended to be lighter than viable ones; this model included diet and temperature as fixed factors. Since the above analysis indicates that meconium retention significantly inflates estimates of dry mass of inviable samples, this model included only specimens with no evident retained meconium. In the resulting model (Wald *C* ^2^ = 316.74, 338 observations, 2 blocks, *p* < .0001), inviable specimens (marginal mean ± delta‐method *SE*: 0.0674 ± 0.0034 g) were found to weigh an average of 7% less (marginal mean ± *SE*: 0.0674 ± 0.0034 g) than the viable specimens (marginal mean ± *SE*: 0.0725 ± 0.0008 g; *C* ^2^ = 3.99, *df* = 1, *p* = .046); effects of diet (*C* ^2^ = 188.43, *df* = 3, *p* < .0001) and temperature (*C* ^2^ = 26.86, *df* = 2, *p* < .0001) on dry mass remained the same as previously observed.

## 4 DISCUSSION

The primary objective of this experiment was to determine whether temperature and diet stressors imposed during butterfly development would influence FA of adult wing traits. The methods used proved sufficient to detect FA. However, analyses failed to identify FA differences among diet or temperature treatment levels. On the other hand, significant differences in viability and mass were found that generally support our predictions. Specifically, the baseline condition (F1) displayed the lowest mortality and the highest posteclosion dry mass (Prediction A); treatment levels with elevated temperature displayed reduced dry mass (Figure 4b; Prediction set B); also as predicted (Prediction set C), dry mass did not differ between treatment levels in which temperature was elevated throughout development versus when only larvae were subjected to temperature increase (Figure 4b). Treatments reared on chemically defined diets resulted in reduced viability (Figure 3a) and dry mass (Figure 4a; Prediction set D) compared to the baseline, with the greatest reduction occurring in the low‐protein diet (Prediction set E). Individuals that survived to eclose had higher dry mass than those that did not (Prediction F), which supports the expectation that reduced adult mass is evidence of developmental stress.

A result not consistent with our Prediction set C was the greater inviability found at temperature treatment level 3 (both larvae and pupae subjected to elevated temperature; Figure 3b). Since this outcome had not been observed when larvae/pupae were exposed to this regime during project development (during which time larvae were reared on the Carolina diet), we suggest that this result was caused by an interaction between elevated temperature and diet. Unfortunately, this hypothesis cannot be directly examined with the data, due to the unbalanced treatment design (specifically, that individuals fed the Carolina diet were reared only on temperature level 1).

Also, of course, we did not expect to find that observed FA levels would fail to vary among treatments. One interpretation of this result is that stressful conditions induced higher inviability among lower‐quality individuals. By this reasoning, the individuals that survived such conditions intact were those most able to buffer against environmental perturbations and therefore failed to exhibit elevated FA (Polak et al., 2004). Thus, the effect on FA of experimental stressors may have been masked by the differential survival of high‐ versus low‐quality individuals.

Support is provided for the idea that inviability resulting from increased developmental stress masked treatment effects on FA by the finding that dry mass of specimens was lower in both the diet and temperature treatments predicted to be more stressful. This suggests there may be a critical minimum size that larvae must generally obtain prior to pupation in order to metamorphose successfully and that as environmental stress increases, larvae of lower intrinsic quality have more difficulty reaching the target size needed for development to proceed. In any case, the finding that the dry mass patterns cohere with mortality patterns is strong evidence that the temperature and diet levels selected for this experiment were differentially stressful as predicted.

Comment is warranted concerning the result that significant inviability occurred not just under conditions expected to be stressful, but also in the baseline condition (28%), which we considered to be near optimal for the lab‐adapted stock. The incidence of inviability in this treatment is comparable to other reports of mortality for this species when reared under favorable captive conditions (Kelly & Debinski, 1999; Poston et al., 1977). Also, in multiple conversations with representatives from our supplier, we were told that our baseline rate of mortality was typical and to be expected. Regardless of the causes of this mortality (which might include laboratory artifacts such as handling effects), the incidence of inviability in the baseline treatment was significantly less than all other treatment combinations, a result consistent with the logic that increased developmental stress leads to higher inviability.

There are two major, interrelated implications of these results. First, results indicate that lack of evidence for stressor effects on FA may be hard to interpret in field settings in which rates of pupal death or inviability cannot be readily determined. In turn, this consideration suggests that variation in FA of traits may only be measurable within a prescribed—and possibly quite narrow—range of environmental conditions, that is, where “stress” does not cause inviability, and only in accommodating traits (e.g., wing traits cannot be measured in individuals that undergo incomplete eclosion, but it is likely that leg traits might be). This range would have to be determined on a population‐by‐population basis (Bilsma & Loeschcke, 2005), which would decrease the practicality of using FA to measure environmental stress.

An additional issue concerns the identification of traits suitable for inclusion in measures of individual (composite) FA. Such traits should possess several properties, including (a) developmental independence of expression; (b) similar, low levels of measurement error; (c) variation in response to applied stressors; and (d) relative ease of accurate measurement. Much effort was devoted at the outset of this study to identifying traits that met these criteria; nevertheless, in the end, the three selected traits failed to display similar measurement errors. In the absence of copious preliminary data, it remains unclear how to identify suitable traits with a reasonable amount of effort.

Laboratory studies such as this one can be faulted for artificiality of conditions (Fischer et al., 2011; Kingsolver et al., 2009), as well as failure to achieve optimal methodological design, and our experiment is not immune to these criticisms. One limitation here was our failure to achieve a fully balanced design: the inclusion of elevated temperature treatment levels reared on the proprietary diet would have provided greater opportunity to elicit increased expression of FA and, if successful, results might have provided more direct evidence on the relationship between FA patterns and mortality patterns. Had we tracked the development time of each subject, we might have had greater ability to interpret the dry mass results, particularly if we measured the duration of the fifth instar during which much food consumption and growth occurs (Poston et al., 1977; CTS, personal observation) (however, inclusion of this variable here would have compromised our ability to closely control treatment temperature regimes). In nature, ectotherms do not experience constant temperatures (Overgaard & Sørensen, 2008) or chemically defined diets, and several studies have shown that conclusions drawn from lab experiments with constant temperature and controlled diets are not supported by field studies (Clissold et al., 2013; Clissold & Simpson, 2015; Ketola & Saarinen, 2015; Kingsolver et al., 2009), where organisms experience opportunities to exert microhabitat preferences (and thereby increase temperature homeostasis within a preferred range; e.g., Harris et al., 2015; Harrison & Fewell, 1995; Jugovic et al., 2017; Scheffers et al., 2014) and diet choices (Behmer, 2009; Gamberale‐Stille et al., 2014). Possibly, the incorporation in our experiment of a very short‐term temperature stressor during development would have elicited FA (rather than increase mortality, as we contend), since some ectotherms might be able to cope better with short‐term stressors than sustained elevated temperature (e.g., Anderson et al., 2003; but see Fischer et al., 2014 for a contrasting outcome). However, given that our goal was decidedly not to develop procedures for measuring FA in wild populations of *V. cardui*, but rather to use a small ectotherm as a lab model to examine feasibility of developing robust measures of FA that vary in response to imposed stressors, none of these failures calls our results into question or offsets the points that they illustrate. The very fact that organisms are not passive members of natural environments, but rather exert considerable control over the conditions that they experience, adds another layer of complexity to interpreting FA results in the field, namely that of accurately measuring the conditions that individuals actually experience. This consideration reinforces the results of our study, which collectively suggest that FA is difficult to use as a tool to detect the presence of impactful environmental perturbations in nature, especially in small organisms such as insects.

## CONFLICT OF INTEREST

None declared.

## AUTHOR CONTRIBUTION

**Cole Symanski:** Conceptualization (lead); Data curation (lead); Formal analysis (lead); Investigation (lead); Methodology (lead); Writing‐original draft (lead); Writing‐review & editing (lead). **Richard A. Redak:** Conceptualization (supporting); Formal analysis (supporting); Funding acquisition (lead); Resources (lead); Writing‐review & editing (supporting).

## Data Availability

Data will be available via the Dryad Digital Repository at https://doi.org/10.5061/dryad.w0vt4b8pn.
